# Motor Skills Enhance Procedural Memory Formation and Protect against Age-Related Decline

**DOI:** 10.1371/journal.pone.0157770

**Published:** 2016-06-22

**Authors:** Nils C. J. Müller, Lisa Genzel, Boris N. Konrad, Marcel Pawlowski, David Neville, Guillén Fernández, Axel Steiger, Martin Dresler

**Affiliations:** 1 Donders Institute for Brain, Cognition and Behaviour, Radboud University Medical Centre, Nijmegen, The Netherlands; 2 Centre for Cognitive and Neural Systems, University of Edinburgh, Edinburgh, United Kingdom; 3 Max Planck Institute of Psychiatry, Munich, Germany; Nathan Kline Institute and New York University School of Medicine, UNITED STATES

## Abstract

The ability to consolidate procedural memories declines with increasing age. Prior knowledge enhances learning and memory consolidation of novel but related information in various domains. Here, we present evidence that prior motor experience–in our case piano skills–increases procedural learning and has a protective effect against age-related decline for the consolidation of novel but related manual movements. In our main experiment, we tested 128 participants with a sequential finger-tapping motor task during two sessions 24 hours apart. We observed enhanced online learning speed and offline memory consolidation for piano players. Enhanced memory consolidation was driven by a strong effect in older participants, whereas younger participants did not benefit significantly from prior piano experience. In a follow up independent control experiment, this compensatory effect of piano experience was not visible after a brief offline period of 30 minutes, hence requiring an extended consolidation window potentially involving sleep. Through a further control experiment, we rejected the possibility that the decreased effect in younger participants was caused by training saturation. We discuss our results in the context of the neurobiological schema approach and suggest that prior experience has the potential to rescue memory consolidation from age-related cognitive decline.

## Introduction

Many aspects of memory formation decline across the lifespan[1,2]. For procedural memory, the consolidation phase is most notably affected: While younger adults demonstrate enhanced motor performance in a newly learned procedural task after a night of sleep, older subjects do not show such offline consolidation improvements after a phase of memory consolidation[3–9]. However, learning does not happen in isolation; almost every new information or procedure we learn relates to previous experience. Prior experience in the form of motor skill training or expertise helps to maintain motor performance across aging in different fields: experts in fine mechanics such as goldsmiths or watchmakers with at least 10 years of experience show a smaller age-related decline in different motion parameters[10]; and experienced pilots show slower age-related decline in their flight simulator performance compared to less-experienced pilots[11]. Similar effects of expertise have been observed in typists[12] and piano players[13], suggesting that acquired motor skills exert protective effects against age-related decline for expertise-related procedures.

In this study, we investigated whether previously acquired motor skills enhance procedural learning and memory consolidation in different age groups. In a sample of 128 healthy participants, we used a well-established motor learning task that requires sequential finger tapping similar to piano playing[14,15]. After a night of sleep, participants had to perform a retest on the same task, thereby testing offline memory consolidation. As already middle-aged adults appear to experience a decline in motor memory consolidation[6], half of our recruited participants were below the age of thirty years (from here on referred to as ‘younger’) while the other half was between thirty and seventy years (from here on referred to as ‘older’). Half of all participants had extensive experience in piano playing, whereas the other half was not experienced in manual instruments or professional typing. To control for potential effects of more general intellectual abilities on memory consolidation[16–18], we recruited half of our sample among highly intelligent individuals. Finally, to control for possible gender effects[19,20], half of all participants were female and half male. Our main hypotheses were that piano experience had a positive, whereas age had a negative effect on motor learning and consolidation. Potential influences of the control variables intelligence and gender were tested exploratively.

## Material and Methods

### Participants

In the main experiment, we tested 128 participants (64 female, mean age: 34.13 yrs, range: 18–69 yrs). In detail, we included 32 participants (16 female, mean age: 34.7 yrs, range: 21–62 yrs) without considerable experience in playing piano or other manual musical instruments (maximal lifetime experience of 50 hours of manual instrument use; no professional typing); and 32 participants (16 female, mean age: 34 yrs, range: 18–60 yrs) with at least 500 hours of piano training. As a high-intelligence control group, we included 64 members of the Mensa society, which demands performance above the 98^th^ percentile in a standardized intelligence test as admission criterion. 32 of these participants (16 female, age mean age: 33.8 yrs, range: 18–60 yrs) had no or negligible experience in playing piano or other manual musical instruments (maximal lifetime experience of 50 hours; no professional typing), whereas 32 participants in this group (16 female, mean age: 34 yrs, range: 18–69 yrs) had at least 500 hours of piano training. Within any of these subsamples, half of the participants were above the age of 30 years. The full sample in our main experiment hence represented a systematic variation of the factors *piano experience*, *age*, *intelligence* and *gender* (see S1 File details about the balancing of factors between groups). Intelligence and gender were used as controlling factors whereas *age* and *piano experience* were of primary interest. The study was approved by the ethics committee of the University of Munich, procedures were carried in accordance with the approved guidelines and all participants gave written informed consent. During screening by an experienced psychologist, participants reported no history of psychiatric, neurological or sleep-related disorders or drug abuse; no night shifts or transmedian flights during the last month; and no nicotine consumption of more than 5 cigarettes per day. Participants were instructed to refrain from drug intake including alcohol, restrict their caffeine consumption to 2 cups of coffee per day, and follow their habitual sleep patterns during the time of the study.

### Procedures

We used an established sequential finger-tapping task[14,15] consisting of a learning phase followed by a delayed test phase. With their non-dominant hand, participants had to repeat a five digit sequence (4-1-3-2-4) on a computer keyboard. The sequence had to be tapped as accurately and quickly as possible during each 30s trial. As performance measurement we used the amount of correct sequences produced in each trial. Between two trials participants had 20s of rest. The learning phase was performed in the morning between 08:00 and 12:00 and had 12 trials. The test took place 24 hours later and included three trials. Before starting the test on the second day participants were asked whether they slept normally during the night.

### Hierarchical Bayesian modeling of online learning

To compare the groups in terms of starting performance, learning rate, and training benefit on day one, we used a hierarchical Bayesian model for fitting learning curves to the training data for each participant. Learning rate characterizes how fast the participants reach their learning plateau on day one, whereas training benefit is the difference of sequences between the first and last trial. The learning curve[21] is an power-law model of the form: *Y* = *I* + *C*(1 − *R*^*t*−1^), with *Y* = amount of correct sequences during each trial, *I* = initial performance at trial one, *C* = change in performance during day one, *R* = learning rate and *t* = trial number. The model fits different hyper parameters for each group to optimize the fitting routine for each participant based on their group (S1 Fig). The hierarchical nature of the model also makes it robust with regard to the initial values chosen for the model. The model parameters were estimated using Markov chain Monte Carlo sampling in OpenBUGS[22]. The fitted curve parameters for each subject were then used as dependent variables in a two-way factorial MANOVA with *piano experience*, *intelligence*, *age* and *gender* as fixed factors. By using the learning parameters instead of the curves themselves, we avoided distortions associated with averaging of learning curves[23,24].

### Statistical analysis of offline memory consolidation

Offline memory consolidation was measured as difference between number of correct sequences on the last three trials on day one and all the three trials on day two. We used this score as dependent variable in a two-way factorial ANOVA with *piano experience*, *intelligence*, *gender* and *age* (coded dichotomously below vs. above age 30) as fixed factors. The improvement scores were positively skewed. To increase sensitivity of the ANOVA we applied a square root transform to the scores, thereby reducing skewedness. As a control analysis we repeated the same ANOVA with the difference of the best three trials on day one with the three trials on day two. This controls for the possibility of a drop in performance at the end of day one due to fatigue or lack of motivation. A drop in performance would artificially increase the difference between days suggesting a stronger overnight improvement. To test whether each group showed an overnight performance gain we used one sample t-tests for the four different groups. To investigate the effect of differential amount of piano training we tested association between lifetime piano hours with overnight improvement via Pearson correlations. To confirm that there is a parametric relation of age and offline memory consolidation, we correlated age and the memory consolidation benefit for the piano players and non players separately and tested them for a significant difference using Fishers z-transform.

### Control experiment 1

Aim of the first follow up control experiment was to verify whether the effect we observed in the main experiment required a prolonged window of memory consolidation–in our case 24h. Independent from the main experiment we recruited four different groups including young piano players (n = 20, 10 female, mean age: 22.5 range: 18–27), young non-piano players (n = 20, 10 female, mean age: 22.5 range: 18–28), older piano players (n = 14, 5 female, mean age: 59.21 range: 55–70), and older non-piano players (n = 13, 5 female, mean age: 59.15 range: 55–65). We refrained from testing middle-aged participants; this was done since we observed in the main study that the compensatory effect was strongest for the oldest participants, so excluding the middle participants increased statistical power for the age effect. The same exclusion criteria were used as described in the main experiment. Procedures were identical to our main experiment; however instead of having the second session 24 hours later we only waited 30 minutes in which the participants completed a short version of the culture free intelligence test[25].It is a nonverbal reasoning test in which subjects are required to complete abstract patterns by finding their organizing rules[26]. We used the same two-way ANOVA as described in the main experiment, but with the age variable coded dichotomously based on the groups recruited rather than via a median split. To be consistent with the analysis of the main experiment, we coded intelligence dichotomously using a median split of the IQ scores obtained.

### Control Experiment 2

Only the older participants showed a significant effect of *piano experience* for memory consolidation. However, we hypothesized that an effect for the younger participants might be masked by a saturation effect during training (see discussion); in which case an effect would be visible when using a shorter training phase on day one. Again fully independent of the other samples we included young piano players (n = 30, 16 female, mean age: 22.97, range: 18–32) and young non-piano players (n = 37, 20 female, mean age: 23.68, range: 19–28) for this experiment. Participants were further split into two groups, undergoing either 30 minutes (n = 30, 12 piano players) or 24 hours (n = 37, 17 piano players) of consolidation before retest. The same exclusion criteria were used as described in the main experiment. Procedures were identical to our main experiment; with only a shortened version of the task used: instead of having 12 training trials during day one, participants only had 6 trials on day one and then the three test trials either 30 minutes or 24 hours later. Both groups completed the same intelligence test from the first control experiment after the training trials. Statistical analysis was identical to the main experiment, however due to the shorter task length; the mean of trial 4 to 6 was used instead of the mean of trial 9 to 12 as the endpoint of training. Since we only included a young group, age was not used as a factor in this control experiment. We performed a two-way ANOVA with the factors *piano experience*, *intelligence* (dichotomously coded according to median split), *gender* and *consolidation period* between the two sessions as fixed factors.

## Results

### Online learning

**Fig 1:**
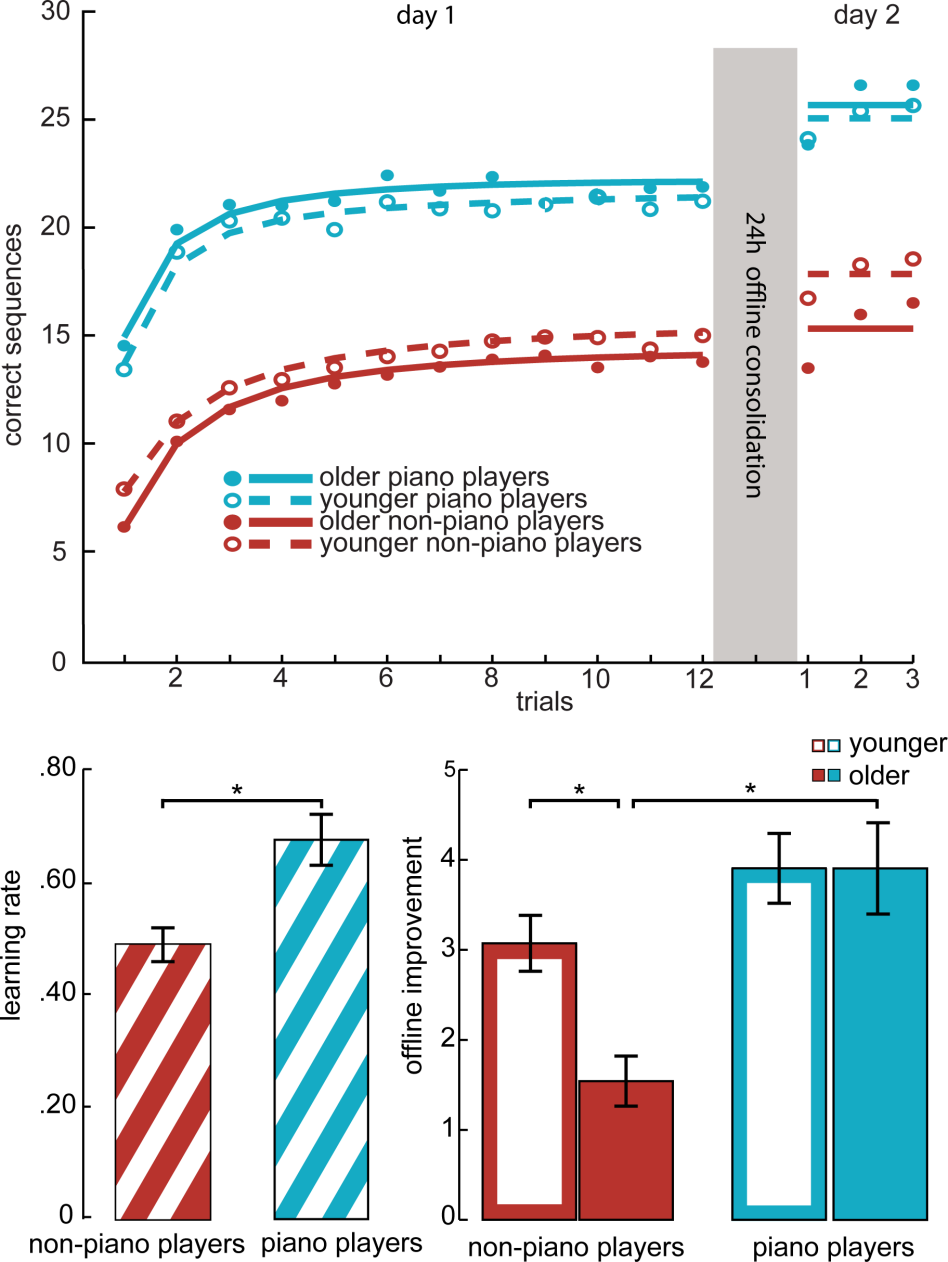
**Top: Performance in the finger-tapping motor task, split for younger and older piano players and non-piano players.** Performance is given by the amount of correctly tapped sequences for each 30s trial. The points indicate the averaged group data, while the curves depict the averaged fitted model for the learning session on day one. For the test session on day two, the line depicts the session mean. **Bottom left: Difference in learning rate.** For illustration purpose 1-R is depicted, the closer to 1 the value is, the faster the participants achieved their plateau performance. Bottom right: Memory improvement from the last three trials of day one to the three trials on day 2. The error bars denote the standard error of the mean; the asterisk denotes a significant difference (p < .05).

To compare the different subgroups of our sample in terms of learning rate, starting performance and training benefit on day one, we used a hierarchical Bayesian model for fitting learning curves to the training data for each participant (S1 Fig). A two-way factorial MANOVA with motor piano experience, intelligence, age and gender as fixed factors and the fitted curve parameters for each subject as dependent variables revealed that piano players demonstrated a higher initial performance (F_1,117_ = 69.86, p < .00001) and an increased learning rate (F_1,117_ = 11.5, p = .0001) during day 1 (see Fig 1). The high intelligence group also showed a higher initial performance (F_1,117_ = 15.82, p = .0001), but no significantly increased learning rate (F_1,117_ = .002, p = .965). No significant effects of age and gender on initial performance (F_1,117_ = .382, p = .538, F_1,117_ = .695, p = .406) and learning rate (F_1,117_ = 1.009, p = .317, F_1,117_ = .892, p = .347) were observed. Furthermore, neither intelligence (F_1,117_ = .011, p = .916), age (F_1,117_ = .015, .903) or experience (F_1,117_ = .028, p = .867) significantly affected the improvement from the first to the last trial during day one.

### Offline memory consolidation

**Fig 2:**
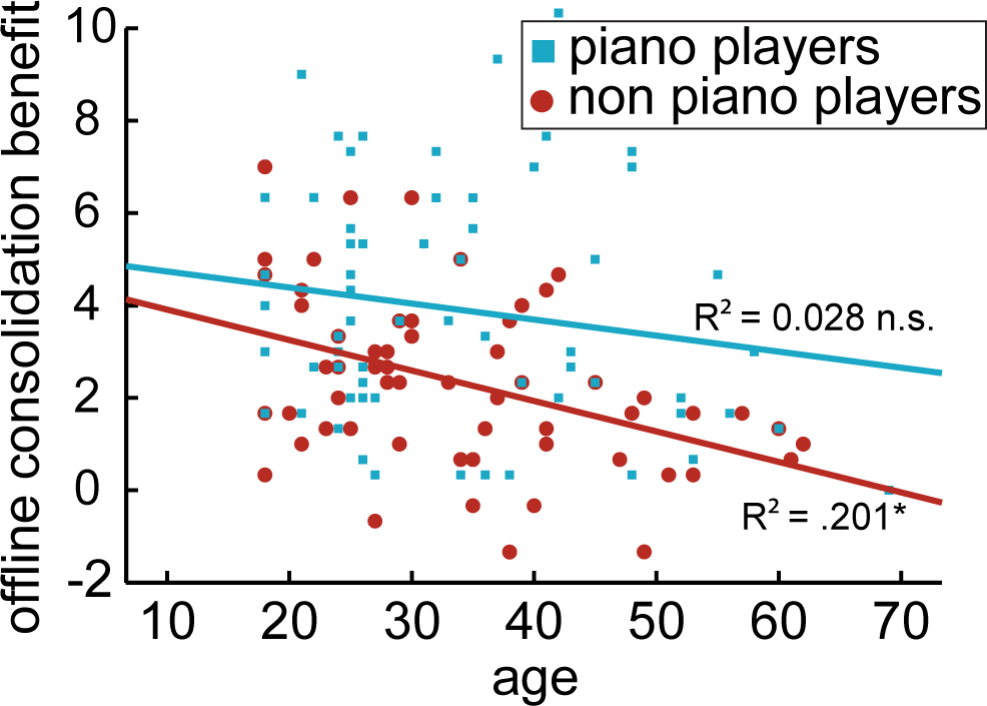
Age-related decrease in memory consolidation. The non-piano group shows a significant decrease in memory consolidation with increasing age (r = -.448, p =. .0002). For the piano players this effect is not significant (r = -.167, p = .187) and weaker as compared to non-piano players (z = -1.73, p = 0.042, one-tailed). This pattern of results indicates that piano experience has a protective effect preventing the usual age-related decline in procedural consolidation. The asterisk denotes a significant difference (p < .05).

The benefit of a 24 hours offline memory consolidation phase was measured as difference between number of correct sequences on the last three trials on day one and all the three trials on day two. All four groups showed significant offline consolidation (younger non-piano players t_31_ = 9.982, p < .00001 younger piano players t_31_ = 10.051, p < .00001 older non-piano players t_31_ = 5.546, p < .00001 older piano players t_31_ = 7.692, p < .00001). A two-way ANOVA with this consolidation score as dependent variable and *piano experience*, intelligence, gender and age (coded dichotomously as younger/older) as fixed factors revealed that piano experience positively influenced consolidation (F_1,117_ = 16.79, p = .00008) whereas age had a negative effect (F_1,117_ = 6.67, p = .011). We observed an interaction between piano experience and age (F_1,117_ = 4.26, p = .0411; Fig 1). Simple effect tests showed no significant impact of the piano experience in the young participants (F_1,117_ = 2.047, p = .155), but a benefit of piano experience for the older participants (F_1,117_ = 18.96, p = .00003). For the piano players effects of consolidation did not diminish with age (F_1,117_ = .132, p = .717), whereas we observed within the non-piano group reduced overnight consolidation for older participants (F_1,117_ = 10.915, p = .001). Piano experience and gender also showed an interaction (F_1,117_ = 4.1, p = .045), with simple effect tests revealing a positive influence of piano skills for females (F_1,117_ = 18.702, p = .00003) but not significantly for males (F_1,117_ = 2.16, p = .144, we discuss this effect in the supplemental discussion—S1 File). Neither intelligence (F_1,117_ = 2.413, p = .123) nor gender (F_1,117_ = .042, p = .838) showed a significant main effect. The negative main effect of age on memory consolidation was confirmed by a negative correlation between age and memory consolidation (r = -0.27, p = .002; Fig 2). Overnight consolidation did not significantly correlate with the amount of hours of piano training (r = .140, p = .262). A control analysis using the best three trials instead of the last three trials of day one did not change the significance of any of the reported findings, indicating that offline improvements cannot be explained by fatigue at the end of day one (see S1 File for the precise statistics). To test whether the increase in offline consolidation of the older piano group was driven by performance differences (Wilhelm et al. 2012), we correlated the mean performance on day one with the offline consolidation score. Neither for the piano (r = .018, p = .324) nor the non-piano (r = –.084, p = .649) older group we observed a significant correlation that would suggest performance differences drive the compensation effect.

### Control experiment 1

**Fig 3:**
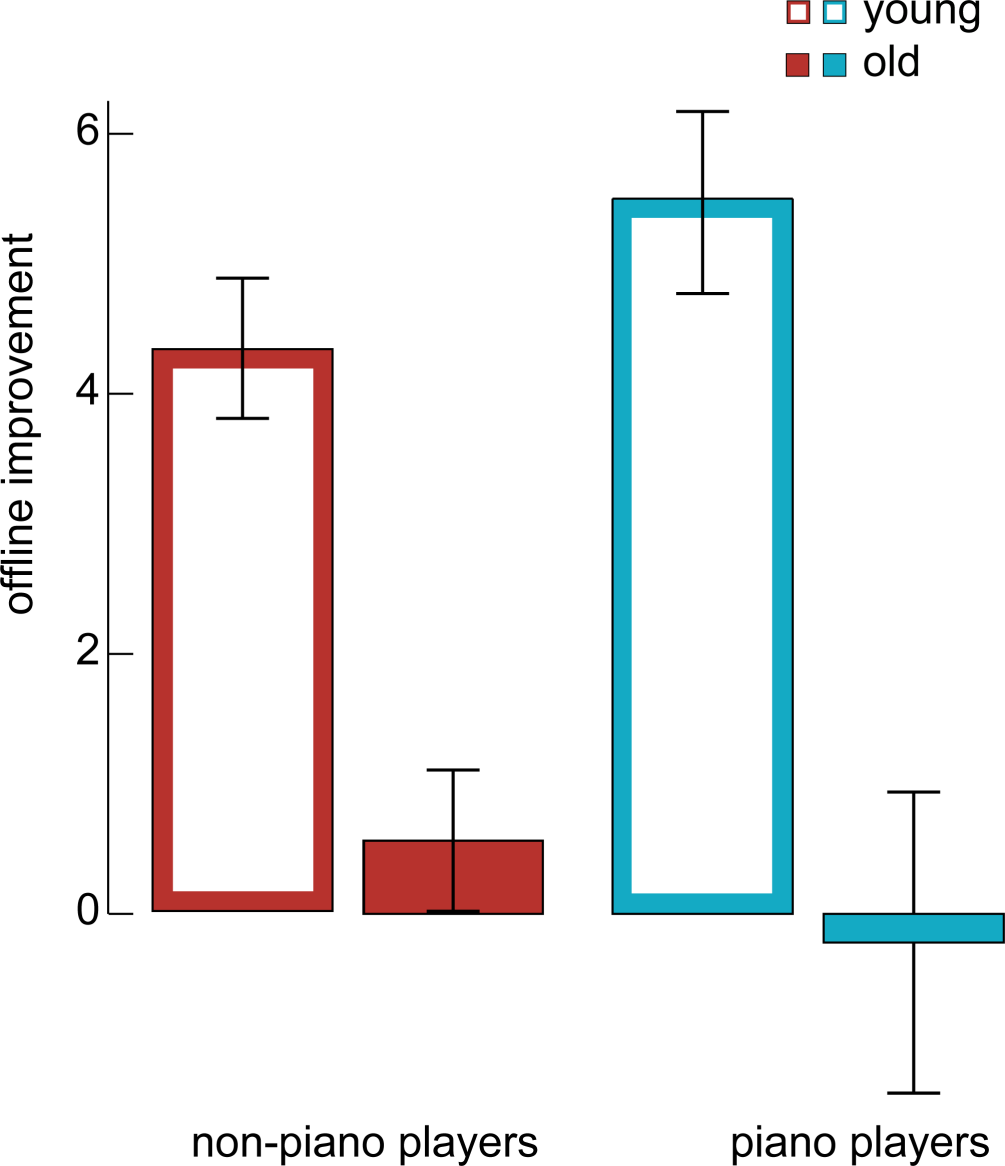
Memory improvement after 30 minutes. In a control experiment we used an identical procedure as the main experiment, however instead of a delay of 24h between the two sessions of the finger-tapping task, memory was tested already after 30 minutes. We did not observe a protective effect of the piano experience on consolidation: old participants showed a significant (F_1,56_ = 28.51, p < .00001) reduction in offline improvement independent whether they were piano players or not. The error bars denote the standard error of the mean.

In an independent sample of 77 participants, we tested whether the effects reported above require a sustained window of consolidation or whether they would already be present after a short offline period of 30 minutes. Younger participants showed a significantly stronger improvement in the finger tapping task after a 30 minute interval than older participants (F_1,56_ = 28.51, p < .00001; Fig 3). Piano experience did not significantly affect the benefit for either group (F_1,56_ = .349, p = .557). Furthermore, neither intelligence (F_1,56_ = .002, p = .962) nor gender (F_1,56_ = .584, p = .448) showed a significant effect; and no significant interactions were observed (p>.1).

### Control experiment 2

**Fig 4:**
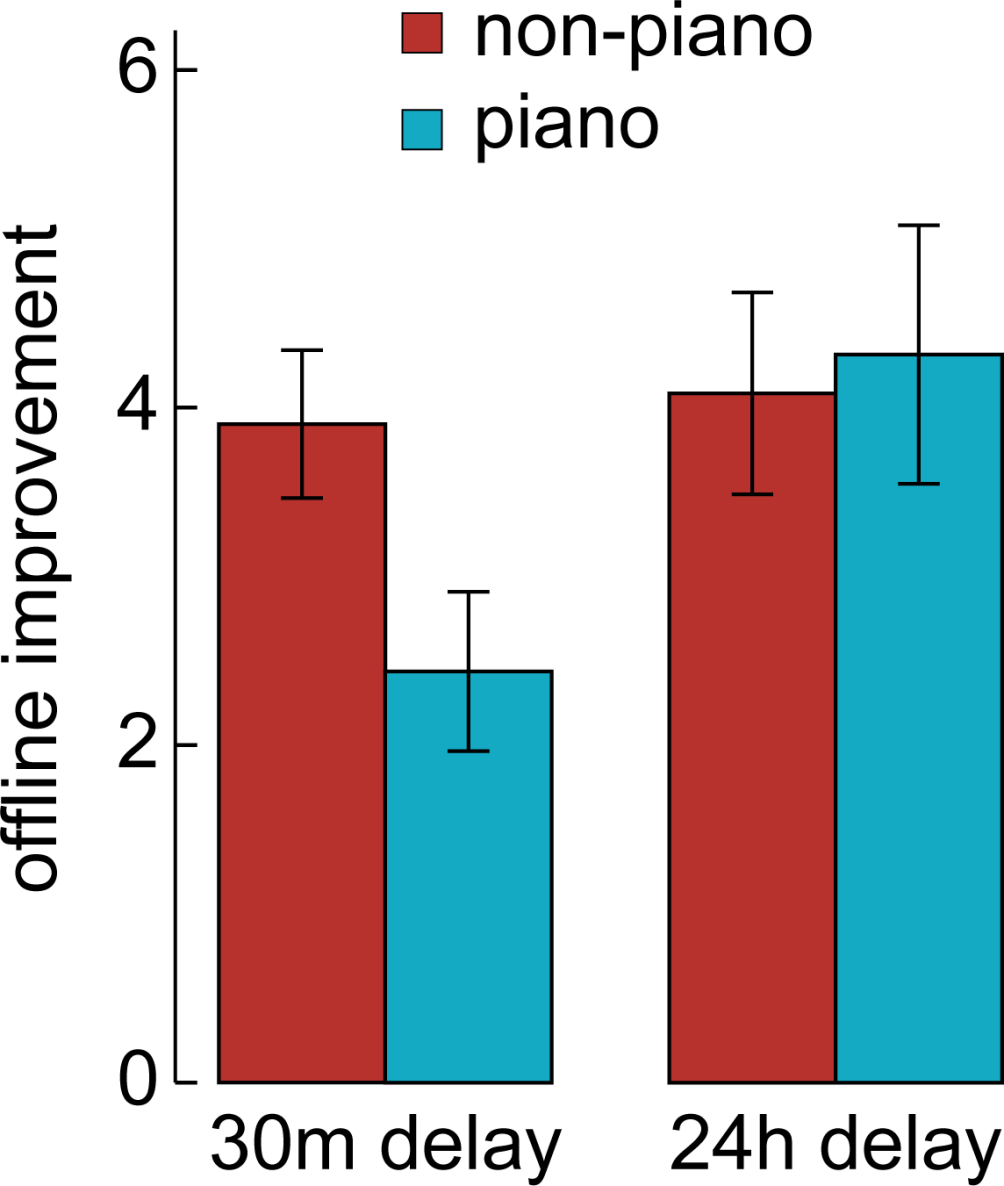
Offline improvement after shorter learning session. In a second control experiment, we tested whether young piano players would show enhanced memory consolidation if the training period is shorter. The procedure was identical to the main experiment, however instead of 12 trials on day one, participants performed only 6 training trials in the finger-tapping task. We did not observe any significant group differences or interactions, independent of whether the delay between training and retest was 30 minutes or 24 hours. The error bars denote the standard error of the mean.

In another independent sample of 67 participants, we followed up on the lack of an effect of piano experience on consolidation in the young age group. We hypothesized that overtraining in the piano group might mask such an. We therefore tested if young piano players would show a benefit of their motor skills with reduced training on the finger-tapping task including only half the number of learning trials. In this control experiment, we did not find a significant effect of piano experience for either 30 minutes or 24 hours of delay on task improvement (F_1,57_ = .827, p>.367; Fig 4). No other factors showed significant differences between groups (p>.05).

## Discussion

Using a sequential finger-tapping task as a model for piano playing, this study demonstrated that the existence of relevant motor experience increased procedural learning speed and overnight memory consolidation. This memory-enhancing effect was selective for the older participants, for whom piano experience protected against age-related decline in offline memory consolidation.

### Memory schemas

Early work of Piaget[27] and Bartlett[28] on cognitive schemas has inspired theories about learning in different fields. When using the schema concept here, we refer to the definition used in current cognitive neuroscience[29–34]: a schema is considered as a previously acquired knowledge structure into which new information can be integrated easily and rapidly.

For declarative memory, different kinds of schemas have been investigated and their memory-enhancing effects linked to the medial prefrontal cortex (mPFC)[31,32,35–37]. The mPFC is further involved in the acquisition of new concepts and generating predictions from them[35], and schema integration during learning is correlated with academic success[37]. Consolidation of new vocabulary is also facilitated by a more extensive prior knowledge of related vocabulary[38], and musical schemas increase consolidation of schema-conformant melodies in respectively enculturated listeners[39].

In the procedural memory domain, the schema theory of discrete motor skill learning by Schmidt[40,41] received much attention. The theory considers schemas as rules that link input parameters of a motion with the outcome of that motion. It especially focuses on what kind of practice schedules would lead to improvements in a variety of sports. Despite some empirical support[42–44], this theory is conceptually of limited use to explain our findings, as it aims to explain under which condition motor learning takes place; however it does not make predictions about the consolidation of newly acquired procedural memories. Besides motor regions such as the primary motor cortex, striatum and cerebellum[45–47], the consolidation of some procedural tasks such as sequential motor learning dependents also on hippocampal processes[48–50]. At least for these tasks, some evidence points towards the adequacy of the neurobiological schema theory: Keyboard-naïve subjects demonstrated transfer effects onto the learning of new sequences compared to the first task exposure on the previous day, suggesting that prior experience facilitates procedural learning[51]. Offline consolidation of this task has recently been linked to hippocampal–medial prefrontal (mPFC)[52] functional connectivity, paralleling similar hippocampal–mPFC connectivity patterns associated with successful consolidation of declarative memories in the presence of a memory schema[32,53]. Together with our results, these studies may suggest that schemas do not only affect declarative memory but extend to the procedural domain as well.

Both perspectives on memory schema have their scope of application; however the study presented here falls in a gap between the two theories. Motor schema theory does not make explicit predictions about memory consolidation, whereas the neurobiological schema theory focusing on memory consolidation has been restricted to the declarative memory domain. Presenting the first evidence for a protective schema effect on procedural memory consolidation, we extend these recent approaches to the procedural domain.

### Prior experience effects on online learning

Piano experience as well as intelligence affected the performance in the first trial on the first day. Additionally, piano players reached their asymptotic performance faster (see Fig 1). All groups showed a similar improvement during the training phase. In task- and keyboard-naïve participants, it has been shown that one learning session with one test following on the next day already facilitates the learning of a second sequence in the sequential finger-tapping task[54]. As piano players show faster finger movements in finger tapping tasks compared to non-musical controls[55], the difference in starting performance was expected. The higher learning rate is congruent with the findings in rats[30]: prior experience enhances learning speed up to the point of single trial learning.

### Prior experience on offline memory consolidation

We observed an enhancing effect of motor skills on memory consolidation for the older, but not younger participants. In effect, piano players did not exhibit the age-related decline in memory consolidation that we observed in the non-piano group (see Fig 1). This was also reflected by a negative correlation between age and memory consolidation in the non-piano group, but not in the piano group (see Fig 2). We interpret this pattern as prior experience–here: piano skills–provides a protective effect against age-related decline of memory consolidation for new but related procedural memories. In other words, task-related experience helped piano players to consolidate newly learned movements as efficiently as young participants. We did not find a relation of the amount of lifetime piano practice with the amount of offline consolidation. It thus appears to be a purely compensatory effect: beyond restoring the amount off offline consolidation, there is no benefit of piano experience. This is consistent with the absence of an effect in the younger population: without an age-related decline, there is nothing to compensate for.

Our results are consistent with previous literature about protective effects of expertise against aging[10–13]. We extend these findings by presenting the first evidence of procedural prior knowledge affecting the consolidation of new but related movements, particularly in older participants. This is relevant as preventing age-related decline does not only affect already known skills, but also learning new procedures in a given domain. For example, an expert pianist does not only want to maintain his or her finger skills, but also the ability to learn and play new unfamiliar pieces. The absence of an effect for the younger participants is consistent with a previous study testing athletes versus non-athletes in multiple tasks including the finger tapping task used here[4]: Baseline performances of athletes was higher than of non-athletes, but there was no significant difference in memory consolidation between groups. The absence of an effect for younger participants will be further discussed in association with the control experiments below.

### Control experiments

One central limitation of our main experiment is that we cannot conclude whether the protective effect on memory consolidation is specific to a prolonged consolidation window–in our case 24 hours–or whether it would already manifest after only a short break of the task. To test this we conducted a control experiment for which we recruited again young and old piano and non-piano players and tested them using a 30 minutes delay instead of 24 hours. Again, we observed the typical age-related decline in memory consolidation, however after 30 minutes we did not observe any effect of prior experience on consolidation (Fig 3). Therefore, we conclude that the protective effect requires a longer window of memory consolidation potentially including sleep.

Indicated by a higher learning rate, piano players reached their behavioral plateau more quickly than the non-piano players. After reaching a plateau, subsequent consolidation of procedural memory does not benefit significantly from additional training[56–58]. Thus, we speculated that young piano players show enhanced memory consolidation compared to non-piano players if the training period is shorter. In a further control experiment we tested this using only 6 trials instead of 12 for training. However also for this shorter training session, we did not observe a significant effect of piano experience after either a 30 minutes or 24 hours delay (Fig 4). This supports the interpretation that prior motor experience does not generally enhance memory consolidation, but rather protects against age-related decline in memory consolidation.

### Limitations

Instead of comparing one young group with a group of elderly we decided to test one younger group below the age of 30 years and a group comprising a broad range from middle-aged to older participants from 30 to 70 years. Thereby, we aimed to contrast a group that is likely not to exhibit any age-related decline in offline consolidation with another group likely showing a continuum of age-related impairments, thus allowing correlational analyses across a broad age spectrum. One consequence of this is that our older group is on average younger than groups investigated in many previous studies[59], rendering direct comparisons difficult. We also cannot draw any conclusions whether the compensating effect we observed also extends into high ages past the ones sampled in our study. Furthermore, we did not acquire any measurements of sleep or brain activity. Therefore we only speculate about the link to the neurobiological schema theory. In our analysis of the main experiment we used age as a dichotomous factor to have a factorial design with equal amounts of data in each cell, potentially limiting the aging related inference one can draw as we did not include a parametric modulation of age. However, complementary we show in Fig 2 that there is also a significant parametric decrease of offline consolidation with. This effect is significantly compensated in the piano players with no evidence for a significant decrease with age.

The evidence for the compensating effect of prior experience in this paper is of correlational nature; we did not actively manipulate the tested motor skills. Thus, we cannot claim any causalities of the explanation presented here, neither can we rule out that there is an additional factor that is specific to the older piano group that is responsible for the effect. One could for example imagine that the piano group is aging more healthily by being cognitively more active. The strongest reason arguing against this account is that the majority of our subjects were still working as only one subject was beyond the age of retirement in Germany. Besides that, it is not evident why any such factor would only hold for the older piano group and not for the highly intelligent older control group.

### Conclusion

Prior experience in playing the piano modulated procedural memory, facilitating acquisition and offline memory consolidation of a sequential finger-tapping task, particularly in older adults. Our results indicate that prior knowledge enhances learning of related movements and protects against age-related decline in memory consolidation. Motor skills acquired through prior experience do not only help to maintain function in a well-trained domain, but also improve consolidation of new aspects in that domain during aging. These results could form a basis for unifying research about the role of memory schemas in both declarative and procedural memory.

## Supporting Information

S1 Fig Learning model. To fit the learning curves, we used a hierarchical Bayesian model. The learning curves are fully characterized by three parameters: I the initial performance, C the change from the initial performance to the asymptote on day one and R the learning rate indicating how quickly asymptotic performance is reached; Y is the performance in terms of correct sequences per trial, t indicates the trials on day one ranging from 1 to 12.[21] The parameters for each subject were drawn from normal distributions; the standard deviation σ and the mean μ for these three distributions were estimated independently for each group. This procedure ensured that no group is favored for the fitting of its final solution being closer to the initial values of the procedure. The model was estimated using the Markov Chain Monte Carlo sampler OpenBUGS[22].
(TIF)

S2 Fig Box plots for the ages in the younger and older group. The red line indicates the mean age; the box contains 50% of the data starting from the 25^th^ percentile ranging to the 75^th^ percentile. The whiskers extend from the 25^th^ or 75^th^ percentile to the farthest data point that is not an outlier (i.e. >1.5 times the length of the box away from either end). In the present plot, no outlier is present.
(TIF)

S1 File Supplemental Methods, Discussion, Results and Refences. The supplemental material contains more details on the sampled ages in the study, the utilized learning model, additional control analysis, and additional discussion of the interaction of gender and piano experience as well as the Appropriateness of the fingertapping task as a model for playing the piano.
(PDF)

S2 File Supplemental Data Control Experiment 1. The raw data from the first Control experiment.
(XLSX)

S3 File Supplemental Data Control Experiment 2. The raw data from the second Control experiment.
(XLSX)
