# Supplementary figures and images for: Motor Skills Enhance Procedural Memory Formation and Protect against Age-Related Decline
Source: PLoS One. 2016 Jun 22;11(6):e0157770. doi: 10.1371/journal.pone.0157770 (PMC4917083; doi:10.1371/journal.pone.0157770)

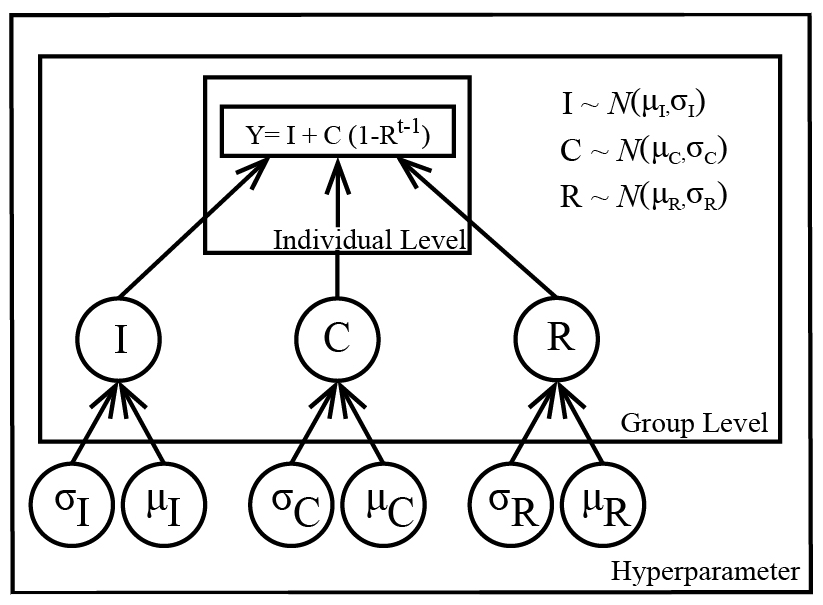

Supplement: S1 Fig — To fit the learning curves, we used a hierarchical Bayesian model. The learning curves are fully characterized by three parameters: I the initial performance, C the change from the initial performance to the asymptote on day one and R the learning rate indicating how quickly asymptotic performance is reached; Y is the performance in terms of correct sequences per trial, t indicates the trials on day one ranging from 1 to 12.[21] The parameters for each subject were drawn from normal distributions; the standard deviation σ and the mean μ for these three distributions were estimated independently for each group. This procedure ensured that no group is favored for the fitting of its final solution being closer to the initial values of the procedure. The model was estimated using the Markov Chain Monte Carlo sampler OpenBUGS[22]. (TIF) [file pone.0157770.s001.tif]

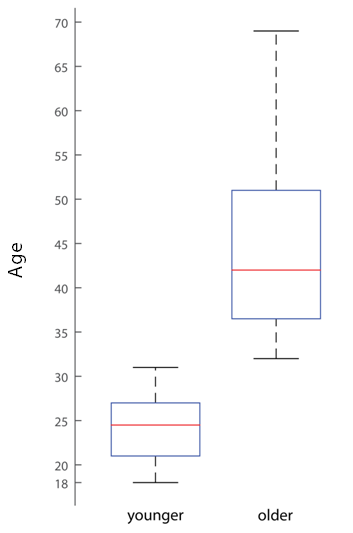

Supplement: S2 Fig — The red line indicates the mean age; the box contains 50% of the data starting from the 25th percentile ranging to the 75th percentile. The whiskers extend from the 25th or 75th percentile to the farthest data point that is not an outlier (i.e. >1.5 times the length of the box away from either end). In the present plot, no outlier is present. (TIF) [file pone.0157770.s002.tif]
