# Supplementary material for: Motor Skills Enhance Procedural Memory Formation and Protect against Age-Related Decline
Source: PLoS One. 2016 Jun 22;11(6):e0157770. doi: 10.1371/journal.pone.0157770 (PMC4917083; doi:10.1371/journal.pone.0157770)
Supplement: S1 File — The supplemental material contains more details on the sampled ages in the study, the utilized learning model, additional control analysis, and additional discussion of the interaction of gender and piano experience as well as the Appropriateness of the fingertapping task as a model for playing the piano. (PDF) [file pone.0157770.s003.pdf]

Supplemental material

Motor skills enhance procedural memory formation and protect against age-related decline

NCJ Müller, L Genzel, BN Konrad, M Pawlowski, D Neville, G Fernández, A Steiger, M Dresler

Supplemental methods

Overview of the age sampling. To balance the main factors for the study, recruited 128 participants; 64 of those being piano players, 64 being no-piano players with not more than 50h experience playing any manual instrument. Furthermore, for both these groups, half of the subjects were members of the German Mensa Society (demanding performance above the 98th percentile in a standardized intelligence test as admission criterion). Each subgroup was sampled to also have an equal amount of male and female participants as illustrated below:

<table>
<tr><td colspan="8">128 participants</td></tr>
<tr><td colspan="4">64 piano players</td><td colspan="4">64 control participants</td></tr>
<tr><td colspan="2">32 normally intelligent</td><td colspan="2">32 Mensa members (IQ>130)</td><td colspan="2">32 normally intelligent</td><td colspan="2">32 Mensa members (IQ>130)</td></tr>
<tr><td>16 f</td><td>16 m</td><td>16 f</td><td>16 m</td><td>16 f</td><td>16 m</td><td>16 f</td><td>16 m</td></tr>
</table>

18-70 years old – matched across cells

To ensure that the factor *age* was balanced across the factors *piano* and *intelligence*, the resulting bins of size 16 were matched in terms of age, ranging from 18-70. This entailed that each of those bins contained both younger and older participants. For a more detailed view on the age distribution please see supplemental Fig S2.

Supplemental results

Analysis of the offline consolidation using the best three trials of day one instead of the last three trials. A two-way ANOVA with this consolidation score as dependent variable and piano experience, intelligence, gender and age (coded dichotomously as younger/older) as fixed factors revealed that experience positively influenced consolidation ($F_{1,117}$=5.694, p=0.019) whereas age had a negative effect ($F_{1,117}$=11.014, p=.001). We observed an interaction between motor experience and age ($F_{1,117}$=4.828, p=.03). Simple effect tests showed no significant impact of experience in the young participants ($F_{1,117}$=.017, p=.898), but a benefit of motor schema for the older participants ($F_{1,117}$=10.499, p=.002). For the piano players, effects of consolidation did not diminish with age ($F_{1,117}$=.132, p=.717), whereas we observed within the non-piano group reduced overnight consolidation for older participants ($F_{1,117}$=15.381, p=.0001). Motor schema and gender also showed an interaction ($F_{1,117}$=4.061, p=.046), with simple effect tests revealing a positive influence of experience for females ($F_{1,117}$=4.502, p=.036) but not significantly for males ($F_{1,117}$=.527, p=.469). Neither intelligence ($F_{1,117}$=.202, p=.654) nor gender ($F_{1,117}$=.980, p=.324) showed a significant main effect.

Supplemental discussion

Interaction of gender and piano experience. We observed that female non-piano players were significantly worse than males with the pattern being reversed within the piano players. Menstrual cycle effects on offline memory consolidation have been reported: women's offline motor memory consolidation profits from a nap only in the mid-luteal phase of the menstrual cycle.[1] We speculate that similar effects might be observed for a whole night of sleep. Therefore, during the task in our main experiment, some woman will likely have been in the mid-luteal phase whereas others were not. This would lead to profits from a window of offline consolidation including sleep only for the subset of the female participants who were in their mid-luteal phase. In turn, on average female participants would have shown a decrease in offline consolidation compared to men. Similar to the compensation effect of the piano experience for aging, this decrease might be compensated in the young female piano players. Hence, it is possible that the piano experience acted compensatingly for the menstrual cycle related average decrease observed in the female participants. However, as we did not control for menstrual cycle or hormonal contraception, this explanation remains speculative.

Appropriateness of the fingertapping task as a model for playing the piano. In our study we used an established finger tapping task as a simple model for playing the piano. As for every model, there are simplifications that are not part of the to-be-modeled entity; however, a large part of the fine motor skills used for the task is similar to piano playing. Since we lack e.g. neuroimaging data of finger tapping and piano playing, unfortunately we cannot demonstrate the appropriateness of the finger-tapping model of piano playing on the level of underlying neurobiology. However, we consider the significant difference in baseline performance on day 1 between piano and non-piano group as a proof of concept for the appropriateness of the finger-tapping model of playing the piano. Given that the focus of our study is on motor learning speed and off-line consolidation, not on baseline performance, this proof of concept is independent from the main outcome variables, and thus not circular. On a more specific level, we would like to point out that the strongest similarity of the finger tapping task is probably not with playing a symphony on the piano but rather with finger exercises piano players would regularly perform during practice. In our study we utilized a modified computer keyboard a more naturalistic situation might be to perform the task on a piano instead. We would expect that in line with previous work the effects we observed might have been even stronger: Repp & Knoblich[2] found in an action observance task that piano players compared to non-piano players showed a strong action direction effect while observing someone using a keyboard; this effect was stronger when a piano was used. Non-piano players did show this pattern.

Supplemental references

1. Genzel, L. *et al.* Sex and modulatory menstrual cycle effects on sleep related memory consolidation. *Psychoneuroendocrinology* **37,** 987–998 (2012).
2. Repp, B. H. & Knoblich, G. Performed or observed keyboard actions affect pianists' judgements of relative pitch. *Q. J. Exp. Psychol.* **62,** 2156–2170 (2009).
3. Manoach, D. S. *et al.* A failure of sleep-dependent procedural learning in chronic, medicated schizophrenia. *Biol. Psychiatry* **56,** 951–956 (2004).
4. Lunn, D., Spiegelhalter, D., Thomas, A. & Best, N. The BUGS project: Evolution, critique and future directions. *Stat. Med.* **28,** 3049–3067 (2009).
